# Mitigation of acyl-homoserine lactone (AHL) based bacterial quorum sensing, virulence functions, and biofilm formation by yttrium oxide core/shell nanospheres: Novel approach to combat drug resistance

**DOI:** 10.1038/s41598-019-53920-w

**Published:** 2019-12-06

**Authors:** Fohad Mabood Husain, Anees A. Ansari, Aslam Khan, Naushad Ahmad, Abdulrahman Albadri, Thamer H. Albalawi

**Affiliations:** 1 Department of Food Science and Nutrition, College of Food and Agriculture Sciences, King Saud University, Riyadh, 11451 Saudi Arabia; 2 King Abdullah Institute for Nanotechnology, King Saud University, Riyadh, 11451 Saudi Arabia; 3 Department of Chemistry, College of Science, King Saud University, Riyadh, 11451 Saudi Arabia; 4 National Center for Nanotechnology and Advanced Materials, King Abdulaziz City for Science & Technology, Riyadh, 11442 Saudi Arabia; 5 Department of Biology, College of Science and Humanities, Prince Sattam bin Abdulaziz University, Alkharj, 11942 Kingdom of Saudi Arabia

**Keywords:** Applied microbiology, Nanostructures

## Abstract

The present study evaluated the efficacy of Y_2_O_3_:Tb (core) and Y_2_O_3_:Tb@SiO_2_ nanospheres (core/shell NSs) against virulence functions regulated by quorum sensing (QS) and biofilm formation in pathogenic bacteria. Scanning electron microscope (SEM) images were used to study the size, shape, and morphology. The images clearly displayed spherical shaped, mono-dispersed particles with narrow size distribution and an average grain size of 110–130 nm. The chemical composition of the samples was determined by using energy dispersive X-ray (EDX) and X-ray photoelectron spectroscopy (XPS). We determined the impact of core and core/shell NSs on QS using sensor strains of *Chromobacterium violaceum* CVO26 and *Pseudomonas aeruginosa* PAO1 in a comparative study. Sub-MICs of core and core/shell NSs substantially suppressed QS-controlled violacein production in *C*. *violaceum*. Similar concentration-dependent effect of sub-MICs of synthesized core and core/shell NSs was observed in the QS-regulated virulence functions (elastase, total protease, pyocyanin production, swarming motility, and exopolysaccharide production) in PAO1. A concentration-dependent decrease (14–60%) was recorded in the biofilm forming capability of PAO1, upon treatment with core and core/shell NSs. Moreover, core/shell NSs were more effective in inhibiting biofilm at higher tested concentrations as compared to core-NSs. The synthesized NSs demonstrated significantly impaired attachment of cells to the microtiter plate indicating that NSs target biofilm inhibition at the attachment stage. Based on these results, we predict that core and core/shell NSs may be an alternative to combat the threat of drug-resistant pathogenic bacteria.

## Introduction

The emergence and spread of multidrug-resistance in bacteria has led to a multi-fold increase in mortality and morbidity globally^1^. Antibiotic resistance is considered one of the biggest threats to global health and is expected to have a huge economic impact^2–4^. The emergence of bacterial strains resistant to most of the currently available antibiotics is alarming^5,6^. On the other hand, there has been no major breakthroughs in the field of new antibiotic discovery since the “Golden era of antibiotic discovery” (1940–1960)^2^. Though some initial success was achieved during the late 60 s to mid-70s, by modification of the already available antibiotics^4^, by 1990, it was clear that the spread of bacterial resistance to antibiotics had far greater pace than the induction of new antibiotics^2,4^. Subsequently, over the past few decades, new technological platforms combining synthetic chemistry and genomics approach also failed to provide even a single agent in the industrial pipeline^3,7^. Thus, the depletion of the antibiotic arsenal and the continuous rise in resistance has led to the exploration of novel antibacterial therapeutics that are refractory to the development of antibiotic resistance mechanisms^2–4^.

Quorum sensing (QS), a global regulatory network, characterized in the majority of bacteria including drug resistant biofilm-forming pathogens, involves the production of extracellular small signaling molecules or auto-inducers (AIs). The subsequent detection of these molecules induces the expression of specific genes, as a function of increasing bacterial population density^8,9^. The expression of QS-controlled genes is involved in the production of virulence factors and plays a key role in bacterial pathogenesis^10,11^. Thus, the disruption of bacterial communication system offers an alternative and possibly safer strategy for effective control of bacterial infections. Targeting QS is advantageous over conventional anti-bacterial strategies, as the former treatment does not involve the suppression of bacterial growth and hence does not exert a selective pressure, which may lead to the development of resistance^12,13^.

In this context, the use of nanomaterials as potential quorum sensing inhibitors (QSIs) against nosocomial pathogens including *Pseudomonas aeruginosa*, is being explored by researchers^14^. Nanomaterials offer several remarkable features, such as large specific surface area, small size, excellent biocompatibility, and in limited concentrations, they are non-toxic and eco-friendly in nature. Among various semiconductor metal oxides, yttrium oxide nanoparticles have attracted growing interest owing to their excellent physiochemical properties^15,16^. They are transparent in the visible region, semiconductor in nature, have high refractive index, large gap band (5.8Ev), high specific surface area and volume, high photochemical stability and excellent biocompatibility at environmental conditions^17,18^. These novel features make them ideal candidates for use in the various biomedical applications^19,20^.

In the present study, we chemically synthesized and characterized the core and core/shell NSs. Scanning electron microscopic images and X-ray photoelectron spectroscopy were used to determine the size, shape, surface morphology and chemical composition of the prepared NSs. In a comparative study, we evaluated the efficacy of core and core/shell NSs against QS mediated virulence factors and biofilm formation in gram-negative pathogens. To the best of our knowledge, this is the first report to investigate the impact of yttrium oxide on the interference of QS and biofilm inhibition. These findings suggest that chemically synthesized metal oxide NSs could be exploited in medical settings as well in the food & beverage industries for removal of microorganisms.

## Methods

### Sample preparation

Y(NO_3_)_3_6H_2_O (99%, BDH chemicals England), Tb(NO_3_)_3_6H_2_O (99%, Alfa Aesar, Germany), Si(OC_2_H_5_)_4_ (TEOS, E-Merck, Germany), and CH_4_N_2_O (Urea) were used directly as received. Milli-Q (Millipore, USA) H_2_O was used for preparation and characterization of the samples. All the analytical grade chemicals were used without further purification from the standard brand of E. Merck Limited, India.

### Synthesis of Y_2_O_3_:Tb@SiO_2_ core/shell nanospheres

Spherical shaped Y_2_O_3_:Tb NSs were prepared using methods described in previously published literature reports^17,18^. An equimolar aqueous solution of yttrium nitrate and terbium nitrate in the ratio of 95:05 was mixed with urea, under constant stirring on a hot plate, at room temperature. The resultant mixture was heated up to 100 °C under reflux for 2 h. The precipitate segregated at the bottom of the flask, which was separated by centrifugation, washed with distilled water, and dried overnight in an oven, at 60 °C. A silica layer was deposited by Stober sol-gel process^18,21–23^. Luminescent NSs (100 mg) were dispersed in an aqueous solution by ultra-sonication for 30 min. These NSs were then separated by centrifugation and further dispersed in an aqueous solution containing 80 mL C_2_H_5_OH, 20 mL H_2_O, and 1 mL NH_4_OH, on a hot plate at ambient temperature. TEOS was introduced slowly into the vigorously stirred solution. The reaction proceeded for 5–6 h at ambient conditions. The precipitate obtained was isolated by centrifugation, washed with distilled water, and dried overnight in an oven at 60 °C.

## Characterization

The morphology of the samples was studied using scanning electron microscope (SEM, JSM-7600F JEOL, Japan) energy dispersive X-ray analysis (EDX), operated at an accelerating voltage 5 kV. The chemical composition was determined using X-ray photoelectron spectroscopy (XPS) using ESCA model VG 3000 with monochromatic Mg Kα line (1253.6 eV) radiation.

### Bacterial strains and growth conditions

*Chromobacterium violaceum* 12472 is a wild-type, purple colored bacterium, which produces violacein in response to cognate acyl-homoserine lactone (AHL) molecules. *C. violaceum* CVO26 is a mutant strain that produces violacein in response to exogenous AHL molecules. QS regulated virulence functions are studied using pathogenic model bacteria *P. aeruginosa* PAO1^24^. All strains were maintained on Luria Bertani or LB broth (15.0 g tryptone, 0.5% yeast extract, 0.5% NaCl) solidified with 1.5% agar (Hi-media). Strains of *C. violaceum* 12472, *C. violaceum* CVO26 and *P. aeruginosa* PAO1 were cultivated at 28 °C and 37 °C respectively.

### Determination of minimum inhibitory concentration (MIC)

Macrobroth dilution method of CLSI was used to determine the MICs of synthesized core and core/shell NSs against the test bacterial pathogens^14^. All assays were performed using concentrations below the MICs, i.e., 1/16 × MIC-1/2 × MIC.

### Violacein inhibition assay

Overnight grown *Chromobacterium violaceum* CV026 (OD_600 nm_ = 0.1) was inoculated in Erlenmeyer flasks containing Luria broth (LB). LB supplemented with C6-HSL (10 µM/L) and the core and core/shell NSs and incubated under shaking for 24 h^25^. Violacein production by *Chromobacterium violaceum* (CVO26) in presence of core-NSs and core/shell NSs was studied using the method described by Husain *et al*.^25^.

### Effect on virulence factors production

#### LasB elastolytic activity assay

The elastolytic activity was determined as described^26^. Briefly, treated and untreated PAO1 was incubated for 16 h at 37 °C. Culture supernatant (100 μL) was added to ECR buffer (900 μL) and left at shaking for 3 h at 37 °C. Centrifugation was performed to remove insoluble ECR, and absorption was measured at 495 nm.

#### Azocasein-degrading proteolytic activity

Proteolytic activity in cell-free supernatants of PAO1 was determined using the assay described by Kessler *et al*.^27^. Briefly, culture supernatants (150 μL) from NSs treated and untreated cultures were added to 1 mL of 0.3% azocasein (Sigma, USA) and incubated at 37 °C for 15 min. Trichloroacetic acid was added to stop the reaction. Absorbance was read at 400 nm.

#### Pyocyanin assay

Pyocyanin was measured by extracting 5 mL culture supernatant of PAO1 with 3 mL of chloroform and then re-extracted in 1 mL of 0.2 M HCl, to get a pink to deep-red colored solution. The absorbance of the extracted solution was measured at 520 nm^28^.

### Swarming motility assay

Swarming motility was determined by point inoculation overnight culture of *P. aeruginosa* (PAO1) at the center of the plate medium consisting of LB broth supplemented with 0.3% agar with or without various sub-inhibitory concentrations (25–200 µg/mL) of core and core/shell NSs^29^. Plates were incubated at 37 °C for 24 h and diameter of swarm was measured.

### Extraction and quantification of exopolysaccharide (EPS)

EPS produced in treated and untreated PAO1 was extracted by centrifugation, and the resultant supernatant was filtered. EPS was precipitated by adding ethanol (100%) to the supernatant and incubating at 4 °C^30^. Method described by Dubois *et al*.^31^ was used to quantify the EPS.

### Assay for biofilm inhibition

Microtiter plate (MTP) assay was employed to assess the effect of the synthesized NSs on biofilm formed by PAO1^32^. Briefly, cells with biofilms grown for 24 h in MTP were stained with crystal violet and quantified by measuring absorbance at 470 nm.

### Cell attachment assay

Core and core/shell NSs (100 μL) were added to 96-well microtiter plates, and culture (100 μL) was added into each well. The MTPs were incubated at 37 °C for 8 h without shaking to allow cell attachment and biofilm development. Following incubation, the modified crystal violet assay was performed to assess biofilm biomass^33^.

### Analysis of *lasB and pqsA* transcriptional activity in *E. coli*

The effect of NSs on the *lasB and pqsA* transcriptional activity in reporter strains, *E. coli* MG4/pKDT17 and *E. coli* pEAL08-2 was measured using the β-galactosidase assay^32,33^. Briefly, quorum-sensing signal molecules (AHLs) from overnight grown PAO1 culture supernatant were extracted with ethyl acetate. Subsequently, 2 mL of reporter strains and 0.5 mL of the extracted supernatant were incubated in a rotatory water bath for 5 h at 30 °C. Further, the mixture was centrifuged, resultant pellet was resuspended in Z buffer (Na_2_HPO_4_ .7H_2_O, 0.06 M; NaH_2_PO_4_.H_2_O, 0.04 M; KCl, 0.01 M; MgSO_4_.7H_2_O, 0.001 M; β-mercaptoethanol, 0.05 M; pH 7.0). Chloroform (200 µL) and 0.1% sodium dodecyl sulfate (100 µL) were added to lyse the cells, and 0.4 mL of O-nitrophenol-β-D-galactopyranoside was also added. Upon appearance of yellow color, the reaction was stopped by addition of 1 mL of 1 M Na_2_CO_3_. Absorbance was read at 420 and 550 nm. Units of β -galactosidase were calculated as 1000 × OD_420_ nm-(1.75 × OD_550nm_)/time × volume × OD_600nm_.

### Statistical analysis

All experiments were performed in triplicates and data obtained from the experiments were presented as mean values. The differences between control and test were analyzed using Student’s *t* test.

## Results and Discussion

### Morphological characterization

**Figure 1:**
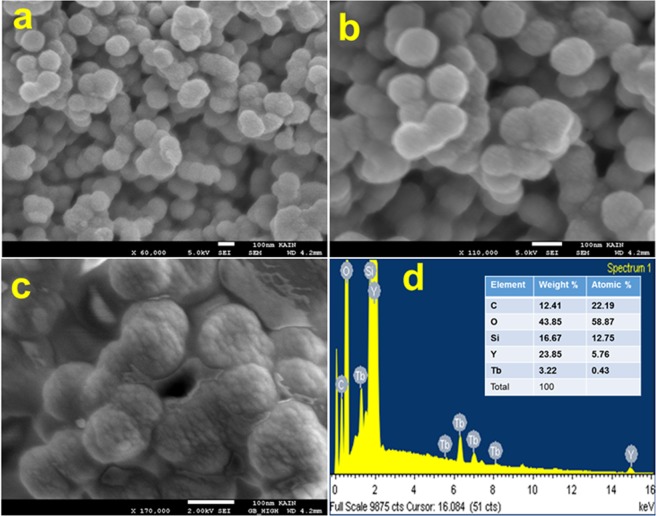
(**a**) Low magnification SEM image (**b**,**c**) high magnification SEM image and (**d**) EDX analysis of core/shell NSs.

Scanning electron microscopy was performed to analyze the morphology of the prepared samples. Figure 1(a–c) demonstrates that the formed core/shell particles were well separated, spherical shaped, uniform, with a rough surface, narrow size distribution, and average grain size of 110–130 nm. SEM micrographs displayed that the particles are interconnected to each other because of the hydrophilic surface. This was expected owing to the grafting of silica layer over the surface of yttrium oxide NSs. The surface was covered with abundant silanol (Si-OH) groups, which easily form a colloidal solution in aqueous media by hydrogen bonding. Due to the presence of hydrogen bonding, they were connected to each other and slightly aggregated. Energy dispersive X-ray analysis was used to monitor the chemical composition of the designed core/shell NSs (Fig. 1d). Figure 1d, shows all elements in the spectrum, including silica (Si), oxygen (O), yttrium (Y), and terbium (Tb), which correspond to the core/shell NSs. This verified the core/shell nanostructure of the sample.

**Figure 2:**
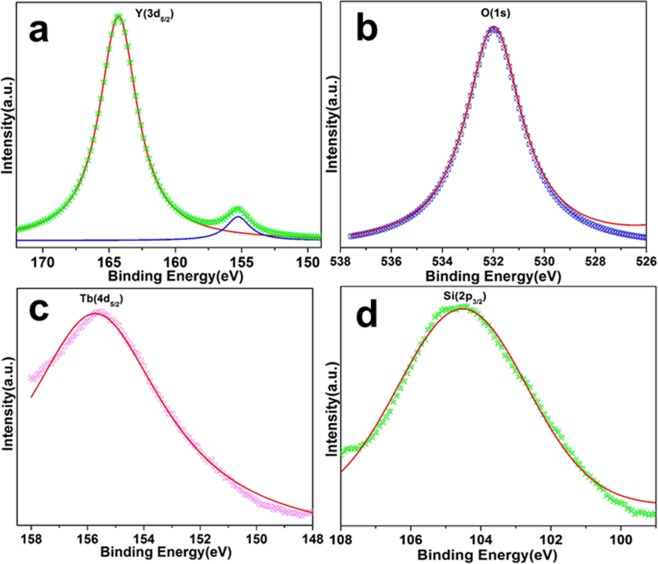
XPS analysis (**a**)Y (3d_5/2_) (**b**) O(1 s) (**c**) Tb (4d_5/2_) and (**d**) Si (2p_3/2_) signals of core/shell NSs.

XPS analysis was carried out to examine the surface chemistry and existence of the Y_2_O_3_: Tb@SiO_2_ core/shell nanostructures. The binding energy signals for Y (3d_5/2_. 164.2 eV), O (1 s, 532.0 eV), Tb (4d_5/2_, 155.7 eV), and Si (2p_3/2_, 104.5 eV), can be seen in Fig. 2(a–d)^34–39^. In accordance with the SEM and EDX observations and literature reports^34,35^, these results inferred that the observed signals arose from the core/shell nanostructures. This provided additional evidence to confirm the successful development of core/shell nanostructures.

### Minimum inhibitory concentration (MIC)

The MIC of core-NSs and core/shell NSs was tested against all test pathogens. The MICs (µg/ mL) of core-NSs were found to be 512, 512 and 1024 against CV12472, CVO26, and PAO1, respectively. Similarly, the MIC of core/shell NSs against all three test bacteria was 256 µg/mL. Sub-MICs were used to assess the QS and biofilm inhibitory potential of the NSs. Our finding is in accordance with those reported against pathogenic bacteria using various nanomaterials^40–44^.

### Anti-QS assay (violacein inhibition)

**Figure 3:**
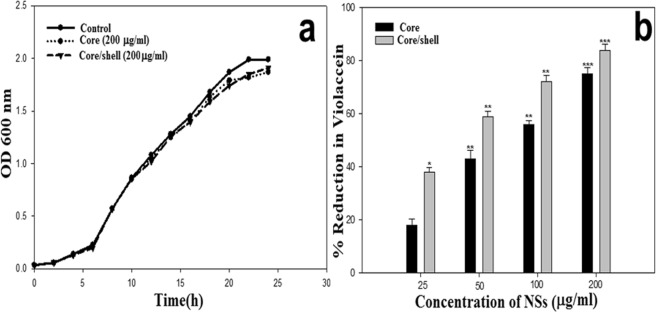
Assay for QS inhibition. (**a**) Inhibition of violacein production in CVO26 by core and core/shell NSs concentrations (25, 50, 100, and 200 μg/mL) was quantified spectrophotmetrically (OD at 585 nm). (**b**) Growth curve analysis of CVO26 at 200 µg/mL concentration of NSs.

Potential quorum sensing inhibitory potential of the synthesized NSs was screened using the biosensor strain *Chromobacterium violaceum* CV12472. Production of a violet-colored pigment called violacein in this strain is regulated by CviIR-dependent quorum sensing system. Therefore, pigment inhibition is indicative of interference with AHL-regulated QS^45^. Violacein levels were quantified by extracting violacein from biosensor strain *Chromobacterium violaceum* CVO26 at sub-MICs of NSs. Colorimetric analysis revealed a substantial drop in violacein production by CVO26 at all tested concentrations of NSs. Core-NSs at sub-MICs of 25, 50, 100 and 200 µg/mL demonstrated 18%, 43%, 56% and 75% reduction in violacein, respectively (Fig. 3a). Similarly, core/shell NSs also interfered with AHL-regulated violacein production in a concentration-dependent manner and 38–84% reduction was recorded (Fig. 3a). The effect of the core-NSs and core/shell NSs (200 µg/mL) on the growth of CVO26 was also examined and no significant difference in cell density was recorded (Fig. 3b). These findings demonstrate that the inhibition of violacein by NSs in CVO26 is growth independent i.e. the growth is not affected. Thus, it is anticipated that the NSs (core & core/shell NS) interfere with AHL-regulated QS signaling. Our findings are validated by reports on anti-QS properties of NSs. Silver nanowires demonstrated 80% decrease in violacein production in CVO26 at 4 mg/mL concentration^46^, while Singh *et al*.^47^ demonstrated 100% pigment inhibition with biogenic silver NSs.

### Inhibition of QS-regulated virulence factors by core and core/shell NSs

**Figure 4:**
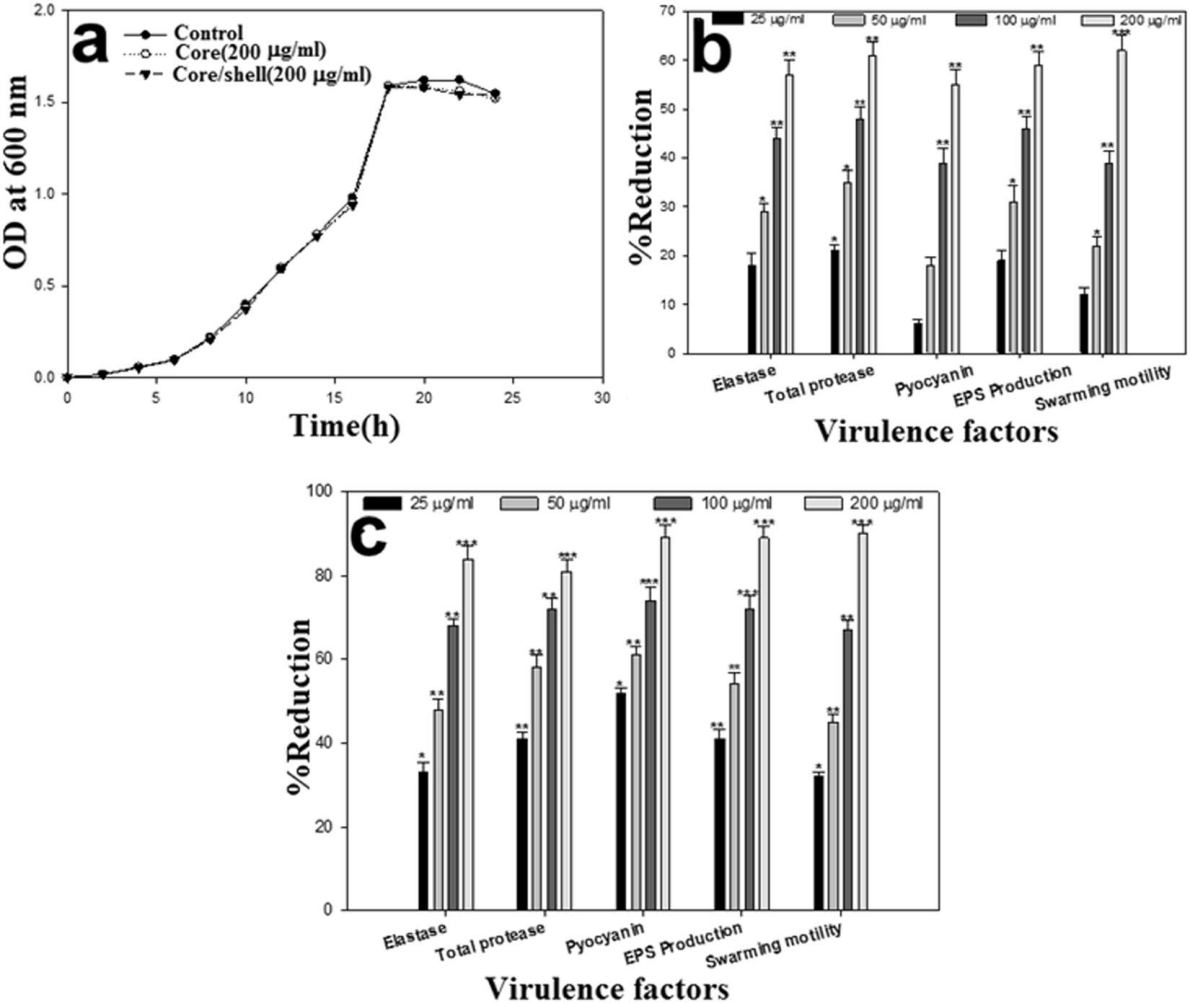
Effect on QS regulated virulence traits of PAO1. (**a**) Growth curve analysis of PAO1 at 200 µg/mL concentration of NSs. (**b**) Effect of sub-MICs of TYO on inhibition of quorum sensing regulated virulence factors. (**c**) Effect of sub-MICs of TYSO on inhibition of quorum sensing regulated virulence factors. Data is presented as mean percent reduction ± SD. ^*^Significance at p ≤ 0.05, ^**^significance at p ≤ 0.005, ^***^Significance at p ≤ 0.001.

Growth kinetics studies of PAO1 revealed no significant change in cell density after treatment with 200 µg/mL concentration of core and core/shell NSs as compared to untreated control (Fig. 4a). Thus, the synthesized NSs do not inhibit the growth of the pathogen. The effect of sub-MIC of synthesized core and core/shell NSs on various QS-regulated virulence functions in PAO1 was also assessed. Core-NSs demonstrated the reduction of all virulence factors in a concentration-dependent manner (25–200 µg/mL): elastase (18–57%), total protease (21–61%), pyocyanin (06–55%) (Fig. 4b). Core/shell NS was found to be more potent in inhibiting the studied virulence factors over the same concentration range. The core/shell NS treated PAO1 cells displayed substantially reduced azocasein degrading protease activity (84%), elastase activity (81%), and pyocyanin production (89%) at 200 µg/mL concentration as compared to untreated control (Fig. 4c).

Protease and elastase enzymes play a major role in the pathogenesis of *P. aeruginosa*, as they are responsible for host tissue degradation. Production of pyocyanin in *P. aeruginosa* is also QS-regulated and this secondary metabolite along with its precursor causes severe toxic effects. In the present study, the production of these virulence traits was reduced significantly at sub-MICs. These findings also support our previous observations on ZnO nanoparticles that reduced the production of elastase and pyocyanin in clinical strains of *P. aeruginosa*^48^. The effect of synthesized NSs on swarming motility and EPS production by PAO1 was also examined. The addition of sub-inhibitory concentrations (25–200 µg/mL) of core-NSs and core/shell NSs demonstrated a statistically significant reduction of swarming motility and EPS production. The reduction in swarming motility by core-NSs ranged from 12–62% while EPS production decreased by 19–59% over untreated control (Fig. 4b). The core/shell NSs demonstrated a significant reduction in swarming migration and EPS at all tested concentrations. The motility of PAO1 was reduced by 32–90% and EPS production was impaired by 41–89% (Fig. 4c). These findings are significant as these two virulence traits play an important part in biofilm formation by *P. aeruginosa*. Motility and EPS are responsible for the successful attachment, development, and maturation of biofilm. Interference with either of the two leads to impaired biofilm formation thereby reducing the resistance of sessile cells to drugs and host immune system. Singh *et al*.^47^ demonstrated reduced migration diameter of PAO1 upon treatment with 25 µg/mL of biogenic silver NSs. This was probably the first report on NS targeting motility and EPS production in PAO1.

### Effect on β-galactosidase activity

The effect of 25–200 µg/mL concentration of core and core/shell NSs on *lasB* and *pqsA* transcriptional activity was examined using β-galactosidase assay. Core and core/shell NSs reduced the *lasB* transcriptional activity by 41% and 65% respectively. Moreover, 55% and 67% downregulation in *pqsA* was also recorded at 200 µg/mL concentration of core and core/shell NSs (Table 1). Pearson *et al*.^49^ reported a positive correlation between AHL concentration and *lasB–lacZ* expression. Our findings correlate well with the above observations, as reduced β-galactosidase activity is indicative of reduced AHL levels and, therefore, reduced expression of the *lasB* gene. Similarly, core and core/shell NSs reduce the transcriptional activation of *pqsA* and inhibit the *pqs* system that controls the production of virulence factors like pyocyanin. These results indicate that the synthesized core and core/shell NSs have broad-spectrum anti-QS activity and are active against both *las* and *pqs* system of *P. aeruginosa*.

**Table 1:** Effect of Core and Core/shell NSs on transcriptional activity of lasR and pqsA.

Concentration (µg/mL)	Core-NSs		Core/shell NSs	
	lasR	pqsA	lasR	pqsA
Control	943 ± 34.6	1042 ± 23.4	943 ± 34.6	1042 ± 23.4
25	859 ± 21.2	881 ± 36.8	799 ± 24.4	724 ± 31.9
50	714 ± 28.8	635 ± 27.9*	680 ± 38.8	503 ± 43.3*
100	601 ± 42.1*	517 ± 38.2*	536 ± 34.6*	389 ± 48.1**
200	556 ± 34.7*	365 ± 37.1**	424 ± 44.3**	344 ± 31.9**

Transcriptional activity was measured via β -galactosidase activity of the lacZ gene fusion products and expressed as Miller Units. *Indicates significance at *p* < 0.05. **Indicates significance at *p* < 0.005.

### Inhibition of biofilm formation

**Figure 5:**
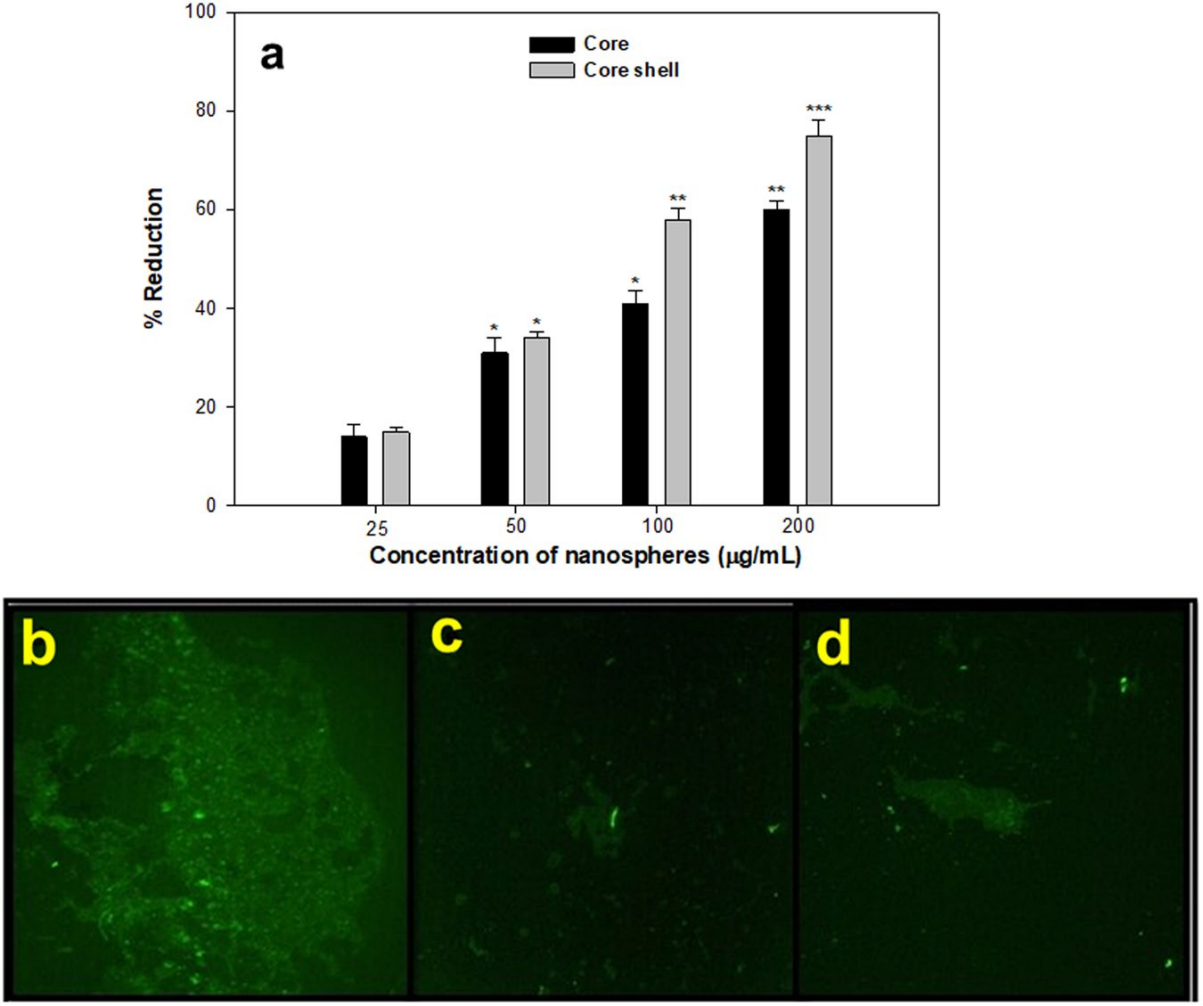
Anti-biofilm activity of NSs. (**a**) Percent inhibition of biofilm formation of PAO1 by sub-MICs of core and core/shell NSs using microtitre plate assay. (**b**) Images of (**a**–**c**) CV-staining light microscope, (**d**–**f**) acridine orange staining CLSM. (**a**,**d**) untreated control; (**b**,**e**) 200 µg/mL core and (**c**,**f**) 200 µg/mL core/shell NSs.

Biofilm inhibition by core-NSs and core/shell NSs was tested using microtiter plate assay. Biofilm formation was reduced by 14–60% by core-NSs in a concentration-dependent manner (Fig. 5a). Core/shell NSs were more effective in inhibiting biofilm at higher tested concentrations as compared to core-NSs. At 25, 50, 100 and 200 µg/mL concentrations, core/shell NSs impaired the biofilm formation by 15%, 34%, 58%, and 75% over untreated control (Fig. 5a). CLSM images confirmed the results of light microscopy displaying major disruption in the architecture of biofilms of PAO1 (Fig. 5b–d). A biofilm is a complex congregation of micro-colonies that is resistant to drugs and plays a key role in the pathogenesis of opportunistic bacteria such as *P. aeruginosa*^50^. Biofilms are therefore interesting drug targets in fight against drug resistance and persistent infections. We recorded a significant reduction in biofilm biomass upon treatment with the core and Core/shell NSs. Our study is in accordance with previous reports that showed the reduction of biofilms in *P. aeruginosa* PAO1 by nanoparticles of zinc oxide^14,51^, tin oxide^52^ and iron oxides^53^, silica nanoparticles^54^ antimicrobials like doxycycline and ceftazidime^29,55^.

### Effect on cell attachment

**Figure 6:**
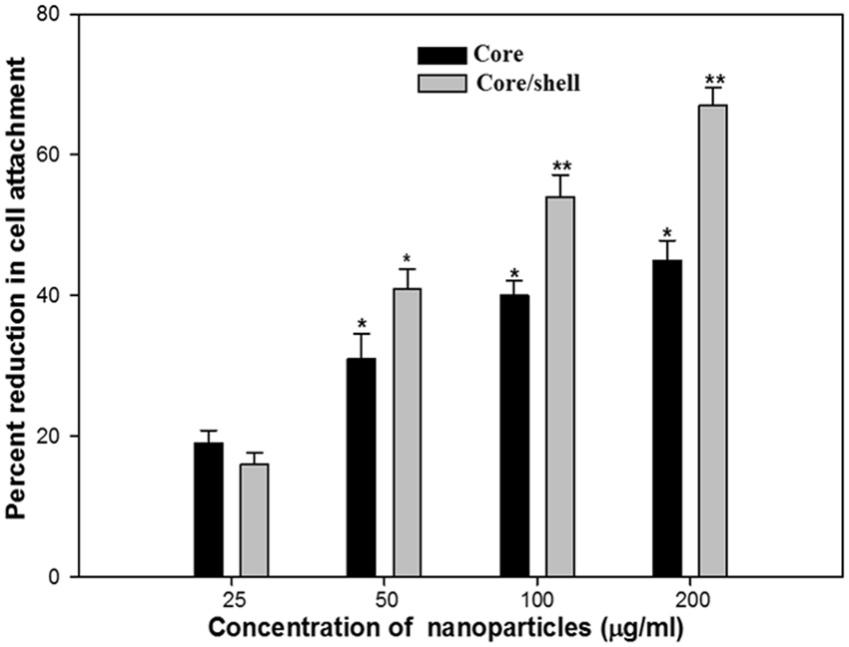
The effect of sub-MICs of synthesized core and core/shell NSs on the attachment of PAO1, expressed as percentage inhibition.

Cell attachment is the first step in biofilm formation and it has been established by previous reports that inhibition of cell attachment to a substrate is easier to achieve than inhibition of the growth of an already established biofilm^56^. Therefore, NSs were examined for anti-attachment property against PAO1. The synthesized core-NSs showed significant inhibition of cell attachment to the microtiter plate. Core and core/shell NSs showed maximum activity of 45% and 67% at 200 µg/mL concentrations, respectively. Core-NSs at concentrations 25, 50, and 100 µg/mL showed 19%, 31%, and 40% inhibition of cell attachment over control. Similarly, 16%, 41%, and 54% inhibition was recorded at lower tested concentrations (25, 50, 100 µg/mL), respectively (Fig. 6). These results indicate that the synthesized core/shell NSs inhibit cell attachment and hence prevent biofilm formation. These core/shell NSs may be used as a coating on surfaces to prevent biofilm formation.

## Conclusion

To conclude, spherical shaped monodispersed core and core/shell NSs were successfully produced by homogeneous urea-based co-precipitation process. The morphology, size, and elemental composition of the samples were characterized by scanning electron microscopy, EDX, and XPS analysis. The present findings highlight the QS and biofilm inhibitory potential of chemically synthesized NSs (Core and Core/shell NSs), which may be exploited to treat drug-resistant infections of *P. aeruginosa* in future. The anti-biofilm activity results clearly indicate that core and core/shell NSs may be helpful in preventing and fighting drug-resistant infections.
